# Topological, chemical and electro-optical characteristics of riboflavin-doped artificial and natural DNA thin films

**DOI:** 10.1098/rsos.171179

**Published:** 2018-02-14

**Authors:** Bramaramba Gnapareddy, Sreekantha Reddy Dugasani, Junyoung Son, Sung Ha Park

**Affiliations:** Department of Physics and Sungkyunkwan Advanced Institute of Nanotechnology (SAINT), Sungkyunkwan University, Suwon 16419, Korea

**Keywords:** DNA, self-assembly, riboflavin, physical characteristics

## Abstract

DNA is considered as a useful building bio-material, and it serves as an efficient template to align functionalized nanomaterials. Riboflavin (RF)-doped synthetic double-crossover DNA (DX-DNA) lattices and natural salmon DNA (SDNA) thin films were constructed using substrate-assisted growth and drop-casting methods, respectively, and their topological, chemical and electro-optical characteristics were evaluated. The critical doping concentrations of RF ([RF]_C_, approx. 5 mM) at given concentrations of DX-DNA and SDNA were obtained by observing the phase transition (from crystalline to amorphous structures) of DX-DNA and precipitation of SDNA in solution above [RF]_C_. [RF]_C_ are verified by analysing the atomic force microscopy images for DX-DNA and current, absorbance and photoluminescence (PL) for SDNA. We study the physical characteristics of RF-embedded SDNA thin films, using the Fourier transform infrared spectrum to understand the interaction between the RF and DNA molecules, current to evaluate the conductance, absorption to understand the RF binding to the DNA and PL to analyse the energy transfer between the RF and DNA. The current and UV absorption band of SDNA thin films decrease up to [RF]_C_ followed by an increase above [RF]_C_. By contrast, the PL intensity illustrates the reverse trend, as compared to the current and UV absorption behaviour as a function of the varying [RF]. Owing to the intense PL characteristic of RF, the DNA lattices and thin films with RF might offer immense potential to develop efficient bio-sensors and useful bio-photonic devices.

## 1. Introduction

After the discovery of double-helical structures and evaluation of their intrinsic functions and properties, DNA molecules have attracted tremendous attention due to the programmability of the DNA base sequences that enables the design of various DNA nanostructures and applicability of DNA molecules to embed functionalized nanomaterials [1–8]. The additional advantages of using DNA in constructing nanostructures and thin films with dimensional precision and fabrication of efficient devices or sensors with functional enhancement are due to the exceptional features of DNA molecules, i.e. efficient self-assembly and molecular recognition [9–15]. DNA can be easily functionalized with various types of ions, drugs, proteins and nanoparticles and this allows explicit functionalities for novel applications in conventional devices or sensors [16–22]. Among various biomolecules, riboflavin (RF) drug molecules have certain advantages for use in medical treatment as well as fabrication of bio-sensors and bio-photonic devices due to the non-toxic, bio-compatible and significant luminescence. RF, which is known as vitamin B2, is essential for living organisms and is available from food and beverages [23]. RF as flavin cofactors participates in reduction and oxidation processes and is metabolized *in vivo* to flavin adenine dinucleotide followed by flavin mononucleotide. As photosensitizing agent, RF is also used to improve blood clotting via platelet treatment and to detect gene mutations caused by UV irradiation [24]. In addition, RF can serve as a biomarker for cancer therapeutics and as a bioactive molecule to sense optical signals due to conjugation characteristics with RF receptor proteins on a cancer cell and significant photoluminescence (PL) characteristics, respectively [25–27].

Although DNA and RF molecules have been studied in terms of their physical, chemical and biological properties, as well as structural characteristics, RF-embedded DNA lattices and DNA thin films have been rarely discussed. DNA lattices (made of artificially synthesized DNA strands) and DNA thin films (extracted from salmon) with RF having its own functionality might provide a significant platform to construct useful devices (e.g. bio-lasers, bio-organic light-emitting diodes and fluorescence sensors) and suggested tuning of sensitive characteristics (by controlling the amount of functionalized materials) [28,29]. In addition, the study of the characteristics of RF-embedded DNA thin films to understand the interaction, binding, energy transfer and conductance between RF and DNA molecules has not been carried out systematically until now.

Here, we fabricate synthetic double-crossover DNA (DX-DNA) lattices [3] (via substrate-assisted growth) and natural salmon DNA (SDNA) thin films (through drop-casting) doped with various concentrations of RF [RF] and study their topological, chemical and electro-optical characteristics. The critical doping concentration of RF ([RF]_C_) in DNA is obtained by analysing the atomic force microscope (AFM) images of the DX-DNA and current, absorbance and PL of SDNA. The physical characteristics of the RF-embedded SDNA thin films (i.e. Fourier transform infrared (FTIR) spectra, current–voltage, absorption and PL measured by an FTIR spectrometer, a semiconductor parameter analyser, a UV–visible absorption spectrometer and a fluorimeter) are discussed in detail.

## 2. Material and methods

### 2.1. Fabrication of riboflavin-doped double-crossover DNA lattices grown on a mica substrate

**Figure 1.**
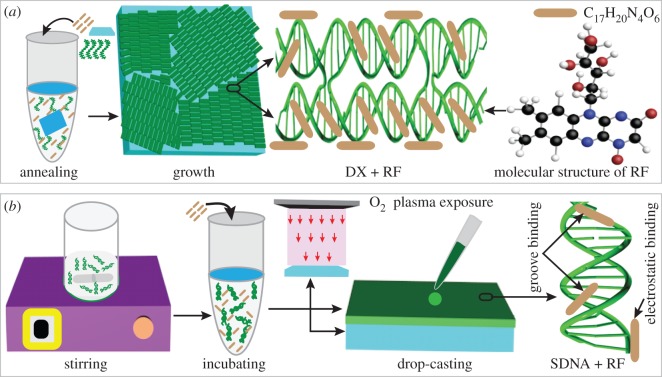
Illustrative representations of sample preparation procedures, RF interactions with DNA molecule, and molecular structure of RF. (*a*) Construction of RF-doped artificially designed double-crossover (DX) DNA lattices on a given substrate by the substrate-assisted growth method. Individual DX DNA strands and RF along with the freshly cleaved mica substrate are added into a test tube in order to grow RF-doped DX DNA lattices on the substrate during the course of annealing. A schematic of a RF-bound DX lattice is shown in the middle. The schematic on the far right shows a simplified (brown-coloured) and a molecular representation of RF. The molecular weight of RF is 376.36 g per mole. (*b*) Fabrication of RF-doped natural SDNA (obtained from salmon fish) thin films formed via drop casting. The substrates are processed using O_2_ plasma to render the substrate hydrophilic which enhances the adhesion of the DNA molecules on the substrate. The sample preparation procedures: dissolving DNA molecules in de-ionized water via magnetic stirring, SDNA incubation with RF for doping, fabrication of the RF-doped SDNA thin film via drop-casting on O_2_ plasma-treated substrate and groove and electrostatic bindings of RF on SDNA duplex.

The synthetic DNA oligonucleotides purchased from Bioneer (Daejeon, Korea) were purified using high performance liquid chromatography. To grow the DX-DNA lattices on mica with various [RF] (Sigma Aldrich, Seoul, Korea), individual DX-DNA strands, freshly cleaved mica having size of 5 × 5 mm^2^, as well as an appropriate amount of RF (i.e. 1, 3, 5, 7 and 9 mM) were added into an AXYGEN-tube. A total sample volume of 250 µl in a 1 × TAE/Mg^2+^ buffer (40 mM Tris, 20 mM acetic acid, 1 mM EDTA and 12.5 mM magnesium acetate) was achieved. The sample test tube was placed in a Styrofoam box with 2 l of boiling water and was then cooled down slowly (about 24 h) from 95 to 25°C for hybridization. During the course of the annealing process, individual DX-DNA tiles were formed in solution and were bound onto a given mica substrate through an electrostatic interaction. We used a final DNA concentration of 50 nM, which was sufficient to be fully covered by DNA on a given substrate (figure 1*a*).

### 2.2. Preparation of riboflavin-doped salmon DNA thin films

To prepare the SDNA solution, an enzyme isolation processed SDNA (Chitose Institute of Science and Technology, Hokkaido, Japan) of 0.1 g dissolved in 10 ml of de-ionized water was placed on a magnetic stirrer at 800 rpm for 10 h at room temperature to obtain 1 weight% (wt%) of homogeneous SDNA solution. To fabricate the RF-doped SDNA thin films, an appropriate amount of RF (i.e. 1, 3, 5, 7 and 9 mM) was added into homogeneous SDNA solution (final [SDNA] of 0.5 wt%) followed by vortex mixing and incubating for a few hours. An RF-doped SDNA solution of 20 µl was drop-cast on a plasma-treated fused silica substrate (size of 5 × 5 mm^2^), and the sample was dried naturally for 24 h (figure 1*b*).

### 2.3. Atomic force microscopy imaging

**Figure 2.**
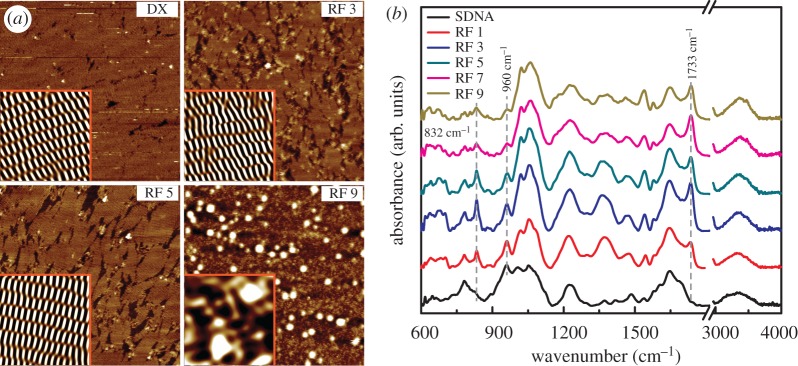
Atomic force microscopy (AFM) images of the double-crossover (DX) DNA lattices and FTIR spectra of the SDNA thin films with various concentrations of RF. (*a*) Representative AFM images (scan size of 1 × 1 µm^2^) of pristine (without RF) and the RF-doped DX DNA lattices with 3, 5 and 9 mM of RF marked as DX, RF 3, RF 5 and RF 9, respectively. Insets in AFM images are noise-filtered images (scan size of 100 × 100 nm^2^) reconstructed through fast Fourier transform to display the periodicity (crystalline phase at or up to RF 5) and aperiodicity (amorphous at RF 9) of the unit building blocks on the lattices. (*b*) FTIR spectra of the RF-doped SDNA thin films in the frequency range from 4000 to 600 cm^−1^. The FTIR absorption spectra of pristine SDNA and SDNA thin films doped with various concentrations of RF are measured to understand the binding mechanism between RF proteins and DNA molecules. The absorption peaks at 832, 960 and 1733 cm^−1^ marked as dotted lines indicate the evidence of RF doping in SDNA thin films.

A mica substrate with RF-doped DX-DNA lattices was placed on a metal disc with the help of instant glue. Thirty microlitres of 1 × TAE/Mg^2+^ buffer was added onto the substrate and another 20 µl of 1 × TAE/Mg^2+^ buffer was dispensed into the AFM tip (NP-S10, Veeco Inc., CA, USA). The AFM images were obtained using a Multimode Nanoscope (Veeco Inc., CA, USA) in the fluid tapping mode (figure 2*a*).

### 2.4. Fourier transform infrared spectroscopy measurement

The FTIR spectra of the RF-doped SDNA thin films with wavenumber in the range of 4000–600 cm^−1^ were recorded using a TENSOR 27 spectrometer (detector: MIR_ATR (ZnSe), Bruker Inc., MA, USA). Thirty-two scans were co-added and averaged with a sensitivity of 4 cm^−1^. The data in the FTIR spectra were analysed by subtracting the background spectrum produced by bare fused silica (figure 2*b*).

### 2.5. Current–voltage measurement

**Figure 3.**
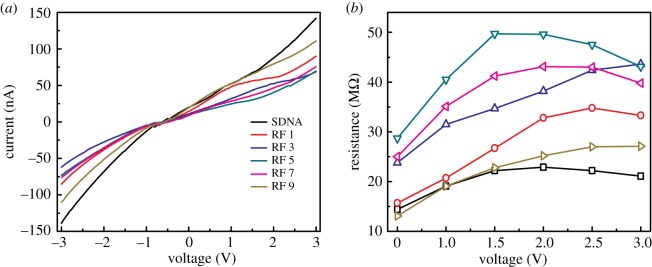
Current–voltage characteristics of RF-doped SDNA thin films. (*a*) Current–voltage curves of the SDNA thin films doped with various [RF] of 0, 1, 3, 5, 7 and 9 mM labelled as SDNA, RF 1, RF 3, RF 5, RF 7 and RF 9, respectively. (*b*) Resistance as a function of fixed voltage (i.e. 0.5, 1.0, 1.5, 2.0, 2.5 and 3.0 V) for pristine SDNA and RF-doped SDNA thin films obtained from the current–voltage curve.

The electrical measurement of the RF-doped SDNA thin film was performed at room temperature using a semiconductor parameter analyser (4200-SC, Keithley Instruments Inc., OH, USA). Silver paste (serving as electrodes) was applied on the surface of the RF-doped SDNA thin film to produce a channel of approximately 1 mm in length (figure 3).

### 2.6. Absorption measurement

**Figure 4.**
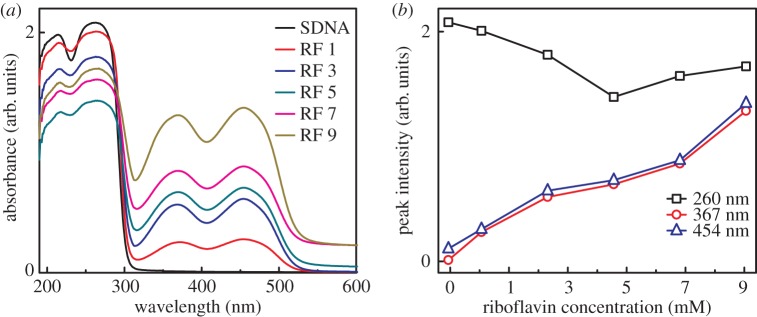
Absorption and absorption band intensity of RF-doped SDNA thin films. (*a*) Variation in the absorption spectra as a function of wavelength for pristine SDNA (without RF labelled as SDNA) and RF-doped SDNA thin films (with dopant [RF] of 1, 3, 5, 7 and 9 mM labelled as RF 1, RF 3, RF 5, RF 7 and RF 9, respectively). (*b*) Changes in the absorption band intensities as a function of [RF] at fixed absorption wavelengths of 260, 367 and 454 nm for pristine and RF-doped SDNA thin films obtained from absorption spectra.

A spectrophotometer (Cary 5G, Varian, CA, USA) was used to conduct the optical absorbance measurement of the RF-doped SDNA thin film on fused silica in the visible and UV regions (wavelength between 600 and 190 nm). The spectrophotometer was equipped with two light sources (a deuterium arc lamp for near-infrared and visible and a quartz W−halogen lamp for UV) and two detectors (a cooled PbS detector for near-infrared and a photomultiplier tube for visible and UV). The spectrophotometer measured the frequency-dependent light intensities of the sample (figure 4).

### 2.7. Photoluminescence measurement

**Figure 5.**
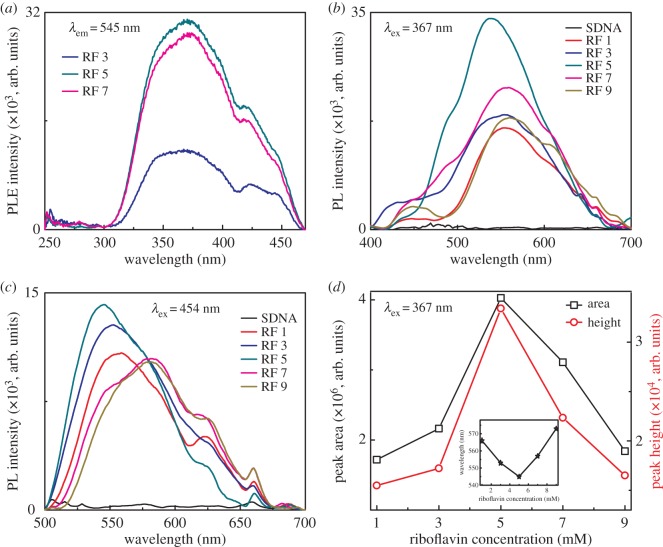
PL characteristics of the RF-doped SDNA thin films. (*a*) PL excitation spectra of the RF-doped SDNA thin films at a fixed emission wavelength of 545 nm. (*b*,*c*) PL spectra of the pristine SDNA and RF-doped SDNA thin films measured at two different excitation wavelengths, i.e. 367 and 454 nm. (*d*) The energetic emission area and height as a function of [RF] in the SDNA thin films obtained from the emission spectra at excitation wavelength of 367 nm using Gaussian fitting. The inset reveals the emission shift as a function of [RF] at excitation wavelength of 367 nm.

The PL and excitation spectra of the RF-doped SDNA thin film were measured at room temperature by using a Xe-arc lamp equipped fluorometer (FS-2, Scinco, Seoul, Korea) with power of 25 W. The excitation spectrum was obtained at fixed emission wavelength of 545 nm, while the emission spectra were measured by exciting the samples at two different wavelengths: 367 and 454 nm (figure 5).

## 3. Results and discussion

Two unit DX tiles were used to construct DX-DNA lattices on the mica substrate using the substrate-assisted growth method [3]. A DX tile was produced by hybridizing two parallel duplexes with two DX junctions cross-linking DNA backbones among two duplexes. Each DX tile had four sticky ends which were preferentially bound to the complementary sticky ends in neighbouring DX tiles, aiding further assembly into DX-DNA lattices. Incubation of RF into DX-DNA lattices was carried out during the course of annealing. By tuning the [RF], the [RF]_C_ at a given concentration of DX-DNA (50 nM) can be obtained through observation of the phase transition from periodic crystalline to aperiodic amorphous structures. The detailed experimental schematics of the DX-DNA lattice fabrication, RF intercalation and molecular structure of RF are shown in figure 1*a*. The individual DX strands, RF, and freshly cleaved mica substrate were added in the test tube in order to grow the RF-doped DX-DNA lattices on the given substrate during the course of annealing.

In addition, a thin film made of natural DNA duplexes extracted from salmon which provided unique and intrinsic physical and biological characteristics was constructed via simple drop-casting (with fixed sample drop-volume of 20 µl) method, as shown in figure 1*b*. The [RF]_C_ at a fixed [SDNA] (0.5 wt%) was predicted by SDNA precipitation in solution above [RF]_C_ and verified by measuring the current, absorbance and PL. Even though we used synthetic DX-DNA lattices (<2.0 nm in thickness) with different [RF] in order to find [RF]_C_ by estimation of morphological changes via AFM, the physical characterizations (i.e. current, absorption and PL) were performed with RF-doped SDNA thin films (thickness approx. 1.5 µm) in order to obtain the significant impact of RF doping with reliable and reproducible results.

To understand the tolerance of the DNA lattice formation with groove and electrostatic binding RFs, the topological characteristics of the DX-DNA lattices with various [RF] (i.e. 3, 5 and 9 mM) were studied. AFM images revealed the tolerance of the DNA lattice formation with RFs, as shown in figure 2*a*. Although the topological features of the RF-doped DX-DNA lattices were mostly similar to pristine DX-DNA lattices (i.e. crystalline phase) up to 5 mM of [RF], they changed dramatically from crystalline to amorphous with an increasing [RF] above 5 mM. [RF]_C_, which was defined as the maximum possible doping [RF] without structural deformation of DX-DNA lattices, was 5 mM. The phase change of the lattices mainly comes from the excess of [RF] (making non-specific binding to DNA) in the sample test tube during the course of annealing.

Figure 2*b* shows FTIR-attenuated reflection spectra for pristine SDNA and SDNA thin films doped with various [RF] (i.e. 0, 1, 3, 5, 7, and 9 mM labelled as SDNA, RF 1, RF 3, RF 5, RF 7 and RF 9, respectively). FTIR spectroscopy was carried out in the frequency range between 4000 and 600 cm^−1^ to demonstrate the specific chemical bond interaction between RF and DNA molecules. Here, we used natural SDNA thin films (having thickness of 1.5 µm) for chemical and electro-optical characterization (e.g. FTIR, current, absorption and PL) due to easy fabrication and manipulation with reliable results. The SDNA thin films without RF showed significant absorption characteristics of water OH stretching (in the range between 3600 and 3000 cm^−1^), and stretching and vibration of nucleobase (1800–1300 cm^−1^) and the sugar and phosphate backbone groups (1250–600 cm^−1^) [30,31].

The FTIR spectra of RF-doped SDNA thin films showed notable absorption band variations (i.e. intensity and position controlled by [RF]) compared to pristine SDNA. While increasing the [RF] in SDNA thin films, two additional notable absorption bands appeared at wavenumbers of 832 and 1733 cm^−1^ (marked as dotted lines in figure 2*b*) which indicated evidence of RF binding into SDNA duplexes. The two bands at 832 (appearing in the phosphate backbone region, 1200 – 700 cm^−1^) and 1733 cm^−1^ (the DNA nucleobases region, 1800 – 1300 cm^−1^) indicate that the RF could be bound with nucleobases as well as phosphate backbone sites of SDNA molecules. Interestingly, the absorption band of C − C and C − O of deoxyribose skeletal motion around 960 cm^−1^ (marked as a dotted line in figure 2*b*) decreased gradually with increasing [RF] and eventually vanished at a higher [RF]. In addition, we observed absorption band broadening of the antisymmetric PO_2_^−^ vibration (around 1220 cm^−1^ occurring on the DNA phosphate backbone), and band shifts from 1005 to 1020 cm^−1^ (due to the C−O deoxyribose stretching mode) and from 1050 to 1060 cm^−1^ (P–O or C–O stretching) while increasing the [RF]. The spectral differences of the SDNA thin films with various [RF] indicated a specific interaction between the RF and SDNA duplexes. These results provide evidence of RF bindings to SDNA indirectly through geometrical and electrostatic interactions [32].

To understand the electrical characteristics of the SDNA thin films with different [RF] (i.e. 1, 3, 5, 7 and 9 mM), the current was measured as a function of voltage (figure 3*a*). Two electrodes made of silver paste with a channel length of approximately 1 mm were placed on the pristine and RF-doped SDNA thin films, and the overall trends in the current of the thin film showed slight nonlinearities by varying the voltage, which might come from the asymmetric electrode contact on the SDNA thin film as well as intrinsic characteristics of the sample. Figure 3*b* shows the resistance (inversely proportional to current) of the SDNA thin films with different [RF] as a function of fixed voltages (i.e. 0.5, 1.0, 1.5, 2.0, 2.5 and 3.0 V). Interestingly, an appreciable increase in the resistance (due to the accumulation of electrically insulating RF) was observed with an increase in the [RF] up to 5 mM (which accidently corresponded to [RF]_C_), and above 5 mM, the RF-doped SDNA thin films showed decreases in resistance (because of non-specific RF binding to SDNA) at almost all voltages.

The absorption spectra (measured wavelength-dependent light intensity) of pristine SDNA and RF-doped SDNA thin films with various [RF] were obtained with a UV–visible spectrophotometer (shown in figure 4*a*). The absorption bands indirectly suggest the binding of RF in the SDNA thin films. The RF in the DNA molecules enhanced the absorption bands around wavelengths of 367 and 454 nm (due to the π–π* transitions from the ground state S_0_ to the two lowest-lying excited states of the singlet manifold states S_1_ (corresponding to 454 nm) and S_2_ (367 nm)) and suppressed intrinsic DNA characteristic peaks at 210 and 260 nm [33–36].

Figure 4*b* shows the variation in the absorption band intensities of RF-doped SDNA thin films as a function of [RF] at three different wavelengths of 260 (a characteristic peak of DNA), 367 (RF) and 454 nm (RF). The characteristic absorption band intensity of the RF-doped SDNA thin film at a wavelength of 260 nm decreased (i.e. hypochromic effect) up to a certain critical [RF] (which corresponded to [RF]_C_ of 5 mM) and increased (hyperchromicity) with an increase in [RF]. Here, hypochromic and hyperchromic effects suggested RF binding to DNA duplexes through a π–π stacking interaction between the RF and DNA and the dissociation of DNA duplexes due to the presence of excess RF (=[RF] – [RF]_C_), respectively. By contrast, the characteristic absorption band intensities of the RF-doped SDNA thin film at 367 and 454 nm showed a monotonic increase (hyperchromicity) of the peak intensities as [RF] increased. Interestingly, a slight band shift (about 4 nm) to a higher wavelength (i.e. bathochromic effect) of the sample at 367 nm was observed. The variation in the absorption band intensities of the RF-doped SDNA thin films as a function of [RF] suggested the presence of RF in SDNA thin films and the interaction of RF with SDNA.

Finally, we demonstrated the PL characteristics of RF-doped SDNA thin films as a function of [RF]. We initially obtained the photoluminescence excitation (PLE) spectra of the RF-doped SDNA thin films at a fixed emission wavelength of 545 nm because the RF molecules are known to show a strong green emission at 545 nm, which is depicted in figure 5*a*. From the PLE spectra, a strong PLE peak at 367 nm in the UV region could be observed. Interestingly, the absorption spectra (figure 4*a*), which were analogous to the PLE spectra, showed strong absorption peaks at 454 nm in the blue region as well as 367 nm in the UV. Consequently, two different excitation wavelengths of 367 and 454 nm were fixed to understand the energy transfer characteristics between the RF and SDNA molecules. The PL was free of a photon upon relief of the electron from the triplet excited state, and the energy transfer arises during internal conversion within the excited singlet state, then from the excited singlet state to the triplet state via intersystem crossing, and then to emissive states [9,31,37].

Figure 5*b,c* shows the PL spectra of the SDNA without and with various [RF] at fixed excitation wavelengths of 367 and 454 nm. Interestingly, the emission spectra of the SDNA thin film with [RF] of 5 mM (i.e. [RF]_C_) showed strong and broad characteristic green emission bands at approximately 545 nm, which could not be observed from the pristine SDNA sample. The PL properties of RF have been extensively studied, and it shows a bright green fluorescence when excited at wavelengths in the range of 360 – 375 nm [27,38]. From the PL spectra of the RF-doped SDNA thin films, the PL intensity was appreciably enhanced by an increase in the [RF] up to [RF]_C_, and it then exhibited a quenching effect with a further increase in the [RF]. The energetic emission peak area and height as a function of the [RF] in the SDNA thin films obtained from the emission spectra at a fixed excitation wavelength of 367 nm using Gaussian fitting of the data are displayed in figure 5*d*. Both the area and height of the emission peaks were initially enhanced by increasing the [RF] up to [RF]_C_ and then decreased with a further increase in the [RF] in SDNA thin films.

The inset in figure 5*d* reveals an emission shift as a function of the [RF] at an excitation wavelength of 367 nm. The emission peak at 545 nm shifted to a lower wavelength (blue shift) with an increase in the [RF] up to [RF]_C_, and it then shifted to a higher wavelength (red shift) with a further increase in the [RF] in the SDNA thin films. The enhancement in the emission and the blue shift of the emission peak at 545 nm up to [RF]_C_ suggested that the RF molecules interacted with the SDNA through groove and electrostatic bindings. The PL quenching effect and red shift of the emission at 545 nm above [RF]_C_ implied that the excess RF molecules might be improperly bound to the SDNA, which resulted in unexpected stress and strain to DNA duplexes, which caused a structural deformation (as shown in figure 2*a*). In addition, we tested the pristine RF (without SDNA) in water, which did not show appreciable emission when compared with the RF-doped SDNA thin film at the same [RF]. Consequently, the emission was notably enhanced when the RF was doped into the SDNA due to suppressing the quenching of RF by water molecules [39].

## 4. Conclusion

In summary, a DNA lattice made of double-crossover tiles and DNA thin film extracted from salmon embedded with various concentrations of RF were fabricated via substrate-assisted growth and drop-casting methods, respectively, and their physical properties were studied. The AFM analysis shows that the critical doping [RF] (=[RF]_C_ of 5 mM) in DNA lattices was obtained through observation of the phase transition from periodic crystalline to aperiodic amorphous structures. To understand prominent intrinsic properties of DNA samples with different [RF], the FTIR, current, absorbance and PL measurements of RF-doped SDNA thin films were conducted. The FTIR spectra indicated the binding strength of RF in DNA through an analysis of the specific wavenumbers of 832, 960 and 1733 cm^−1^. The current curves of the DNA thin film showed slightly nonlinear behaviour by varying the voltage, which might be a result of asymmetric electrode contacts on the RF-doped SDNA thin film as well as intrinsic characteristics of the DNA sample. The absorbance at 260 nm (characteristic band of DNA) decreased with an increase in [RF] up to [RF]_C_ and then increased with a further increase in the [RF]; whereas the absorbance at 367 and 454 nm (characteristic band of RF) increased monotonically with an increase in [RF]. The PL spectra of RF-doped SDNA thin films showed significant enhanced intensities as well as emission shift in the green region due to the efficient energy transfer between RF and DNA. The enhanced and tuneable optical characteristics of RF-doped SDNA thin films might be beneficial for use in various applications, including bio-light emitting diodes, bio-lasers, fluorescence sensors, bio-imaging, photo-sensitizers and bio-sensors in the near future.

## Data accessibility

Our data are deposited at Dryad (http://dx.doi.org/10.5061/dryad.hs518) [40].
